# Laser therapy: palliative care for the Harlequin syndrome?

**DOI:** 10.31744/einstein_journal/2026RC1544

**Published:** 2026-02-02

**Authors:** Felipe Otávio Saraiva França, Guilherme Albuquerque Nicolau, Mariana Machado Lima, Rodrigo Otávio Gama França, Vania Flavia Siqueira Saraiva

**Affiliations:** 1 Centro Universitário Cesumar Department of Medicine Maringá PR Brazil Department of Medicine, Centro Universitário Cesumar, Maringá, PR, Brazil.; 2 Centro Universitário Integrado Department of Medicine Campo Mourão PR Brazil Department of Medicine, Centro Universitário Integrado, Campo Mourão, PR, Brazil.; 3 Instituto de Cardiologia e Odontologia Restauradora Estética Medianeira PR Brazil Instituto de Cardiologia e Odontologia Restauradora Estética, Medianeira, PR, Brazil.

**Keywords:** Laser therapy, Hypohidrosis, Blushing, Harlequin Syndrome

## Abstract

Harlequin syndrome is a rare condition characterized by facial dysautonomia with anhidrosis, hemifacial blush, and other nonfacial manifestations without conventional treatment. This report presents a case study of the potential treatment of Harlequin syndrome using laser therapy. A single-case AB design was used, allowing for a systematic comparison between the baseline and intervention phases. The baseline phase involved data collection during a period without intervention (control condition), that is before laser therap. As evaluation criteria, a clinical assessment of skin turgor, sweating or anhidrosis, and blushing was conducted. Additionally, photographic records were taken before and after treatment, and the patient satisfaction index was analyzed. The Treatment Satisfaction Questionnaire for Medication was used as a model, with adaptations made for specific patient cases. The intervention phase included laser therapy. The patient was an 18-year old female diagnosed with Harlequin syndrome without associated comorbidities. The patient was instructed not to undergo any treatment other than the laser therapy. After the laser therapy, the patient reported improvement in symptoms and satisfaction with the outcome and the therapy. It was concluded that, despite being a rare syndrome with limited available information, laser therapy is a viable and efficient approach for treating Harlequin syndrome.

## INTRODUCTION

Harlequin syndrome (HS) is a rare autonomic disorder characterized by anhidrosis and lack of unilateral facial blush, which can affect the cervical and thoracic regions. Paradoxically, there is a compensatory blush and sweating on the opposite side of the alteration.^(1)^

Harlequin syndrome is usually related to lesions in sympathetic fibers from segments T1-T3 31 of the spinal cord.^(2)^ It is usually idiopathic,^(3)^ however, HS can also occur secondary to brainstem ischemia, mediastinal neuroma, or catheterization of the internal jugular vein. It is a rare syndrome that lacks literature, and the proposed treatments are mainly directed toward causal factors.^(4)^ In this context, laser therapy-based treatment is a possible and effective alternative to palliative care for this syndrome. Infrared laser has an anti-inflammatory, analgesic, and regenerative action, which may support regeneration of the myelin sheath, and is used in the treatment of Bell's paralysis, which has in its pathophysiology a lesion of the myelin sheath. Considering that HS has a similar origin, it may also respond positively to laser therapy.^(5)^

## METHODS

The research met the requirements of Brazil's Resolution 466/2012 CNS and was approved by the relevant research ethics committee. The patient was a female aged 18 years, diagnosed with HS and with no associated comorbidities, and who provided informed consent. A single-case AB study design was used, which made it possible to systematically compare the baseline (A) and intervention (B) stages. The baseline stage consisted of data collection during the non-intervention period (control condition). The intervention stage consisted of laser therapy as a therapeutic procedure. A clinical evaluation of skin turgor, sweating, anhidrosis, and blush was performed as criteria for patient assessment. A before-and-after laser therapy photographic register was performed. Furthermore, a patient satisfaction index was analyzed, using as a model the Treatment Satisfaction Questionnaire for Medication (TSQM),^(6)^ with case-adapted questions for the patient. The questionnaire was named the Treatment Satisfaction Questionnaire for the Patient (TSQP) and comprised seven questions regarding patient satisfaction, efficacy, and convenience of treatment. Each question consisted of four to seven answer options (Portuguese version in Table 1S, Supplementary Material).

For participation in the study, the patient was instructed not to undergo other treatments besides laser therapy and did not show any perceptual-cognitive deficit that prevented her from collaborating in the study. All data were securely stored.

## CASE REPORT

An 18-year-old female reported left hemifacial hyperemia and unilateral sweating on exertion approximately one year previously, with the first medical care occurring in 2020. The patient reported no pupillary changes. In July 2019, she experienced severe torticollis without prior trauma, which improved with the administration of a non-hormonal anti-inflammatory for seven days. Imaging tests were performed to rule out possible major causes.

**Figure 1:**
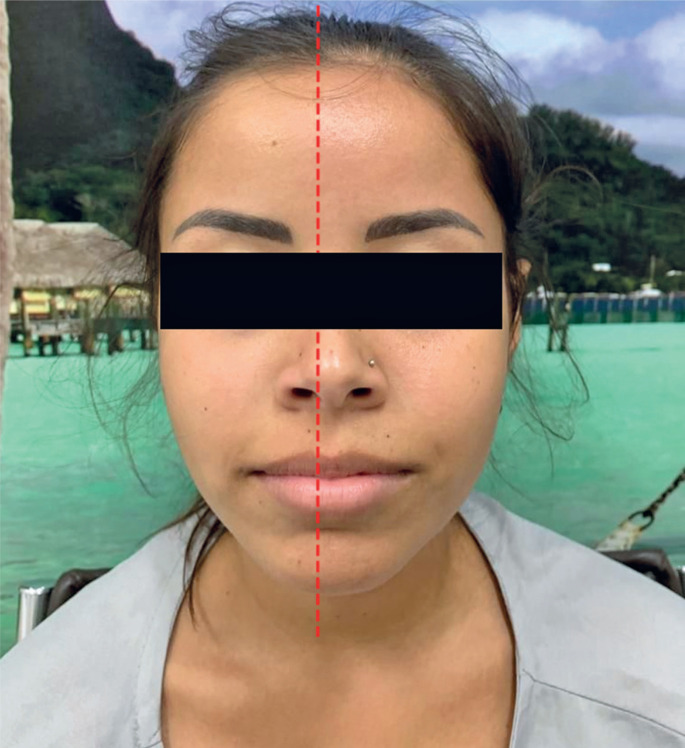
Day 0 – the patient presented anhidrosis and lack of blush in the right frontal region after an ergometric test

At the time of patient assessment, ergometric testing was performed. The test followed the Ellestad protocol, limited to 8-min 13-s of effort to achieve a maximum cardiac rate of 93 % of the predicted rate. The results were normal for ischemia, absence of arrhythmia, and normal chronotropic and pressure responses. During the ergometric test, the patient presented with anhidrosis and lack of blush in the right frontal region (Figure 1).

### Treatment protocol

**Figure 2:**
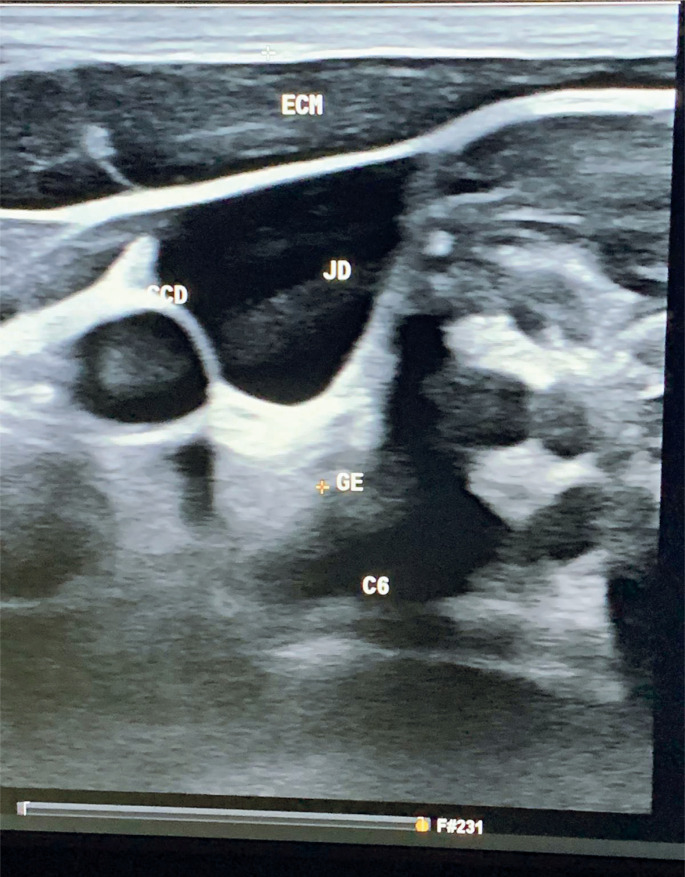
Cervical echo-Doppler mapping of the cervicothoracic ganglion (stellate ganglion)

**Figure 3:**
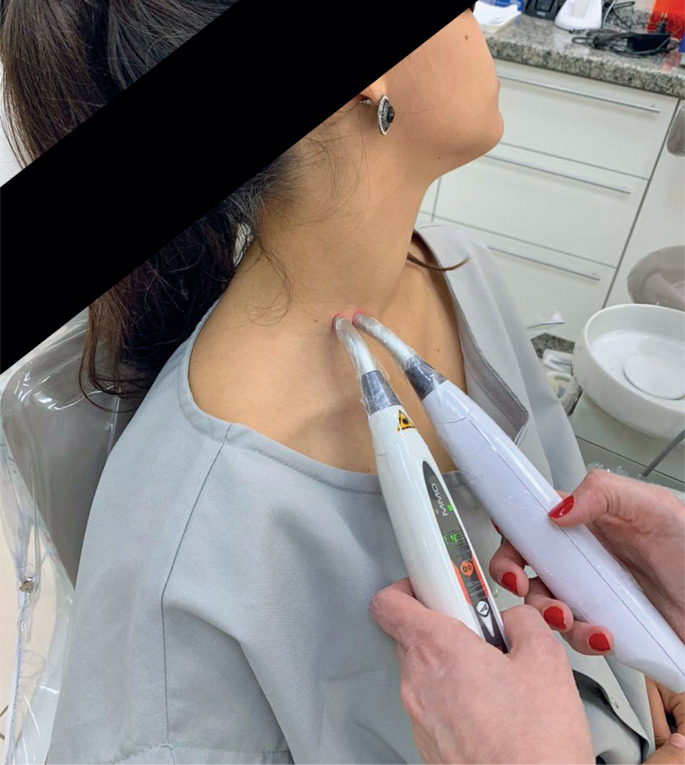
Application of shots from both laser devices on the cervicothoracic ganglion region (stellate ganglion)

For treatment, an authorial protocol was created consisting of 16 applications of 9 J infrared lasers with two Dup MMOPTICS devices used simultaneously, 1cm apart, applied twice in each session. Eight sessions were conducted twice weekly. A diode with a wavelength of 808 nm was used to promote neural repair and analgesia. Laser shots were performed in the right supraclavicular region after mapping with cervical echo-Doppler, tracing the autonomic references to the cervical vertebrae and neck vessels (carotid arteries and jugular veins), and aiming at the cervicothoracic (stellate) ganglion. Shots were performed by a trained professional (Figures 2 and 3).

### Outcomes

**Figure 4:**
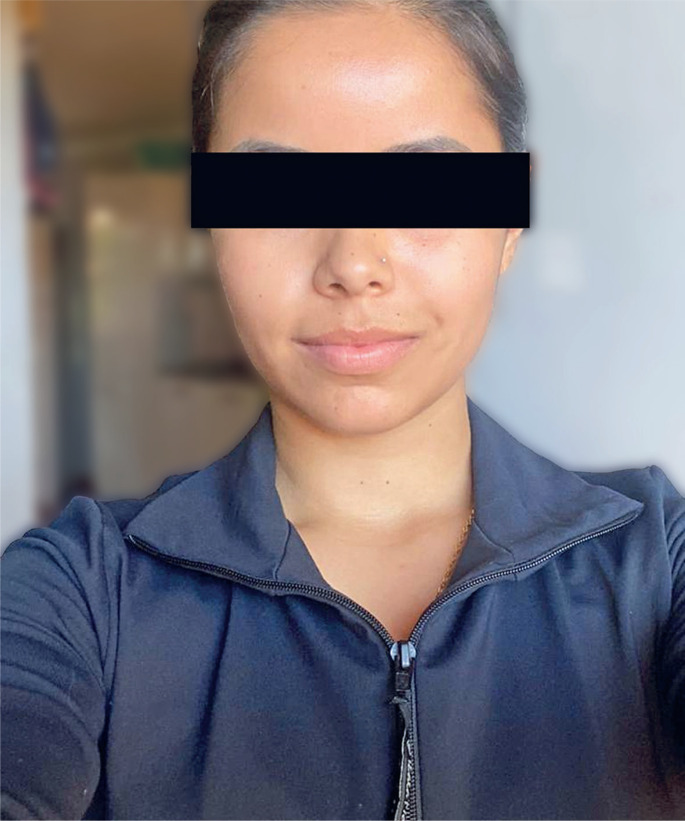
Patient after treatment showing improvement in the symptoms with return of sweating and blushing of the right hemiface

After treatment, the patient showed improvement in her symptoms, reporting sympathetic physiological recovery of the right hemiface, that is, a return of sweating and blushing. Through the application of TSQP, the patient was questioned about the efficacy and quality of the treatment (Table 1). She reported being extremely satisfied with the symptom relief, the time it took to notice the effects, and the results. She also reported that she did not experience pain during the procedure and felt confident about the efficacy of the treatment (Figure 4).

**Table 1:** Questionnaire based on the TSQM model, used for treatment efficacy assessment

How satisfied or dissatisfied are you with the procedure's ability to treat your condition?
Very Satisfied
How satisfied or dissatisfied are you with how the procedure relieves your symptoms?
Extremely Satisfied
How satisfied or dissatisfied are you with the time it takes for the procedure to start working?
Extremely Satisfied
How uncomfortable is the procedure?
I felt no discomfort during the procedure
Overall, how confident are you that this procedure worked well for you?
Very confident
How certain are you that the good things about the treatment outweigh the bad things?
Very confident
Taking everything into consideration, how satisfied or dissatisfied are you with this medication?
Extremely Satisfied

The research was approved by the Research Ethics Committee of Universidade CESUMAR, CAAE: 65588822.0.0000.5539; #5.908.124, and the participant provided written informed consent, authorizing the use of their image exclusively for scientific purposes.

## DISCUSSION

Harlequin syndrome of idiopathic origin is generally caused by insufficiency of the thoracic ganglia of the sympathetic trunk. Abolition of thermoregulation through vasodilatation and hemifacial anhidrosis in HS is caused by homolateral dysfunction of the vasodilator and sudomotor sympathetic neurons that innervate the face, followed by the course of the carotid artery branches.^(7)^ Harlequin syndrome can occur in isolation or can be associated with other dysautonomic disorders such as Horner syndrome, Adie syndrome, or Ross syndrome. In a bibliographic survey by Guilloton et al.,^(8)^ of 118 patients, 44% presented with isolated HS, while 35% presented with HS accompanying other dysautonomic syndromes. HS presents with partial eyelid ptosis, anisocoria, and anhidrosis of the forehead and face. Acute manifestations of symptoms can be an indication of severity and may result from damage to the carotid artery, superior cervical ganglion blockage, inflammatory diseases, drug abuse, and abnormal migration of neural crest neurons.^(9)^

Autonomic cervical dysfunction tends to compromise the patient's'quality of life because it can cause social embarrassment. Correct diagnosis of HS involves a detailed historical survey, physical examinations, autonomic tests, and imaging examinations to rule out secondary diseases. Ipsilateral surgical sympathectomy can be performed, and blockage of the stellate ganglion can be administered repeatedly to patients with this syndrome. Extreme sweating related to HS can also be alleviated by treatment with botulinum toxin, which blocks the cholinergic response of sweat glands.^(7,10)^

In this context, the results achieved with laser application on the stellate ganglion indicate the recovery of peripheral sympathetic neural activity. Contrary to the currently available and commonly disseminated palliative treatments for HS, the proposed treatment in this study did not limit itself to minimizing the contralateral compensatory effects of the face, but sought to recover sympathetic autonomous physiological activity of the affected region. Future follow-up of the patient will allow evaluation of the duration of the effect and the need for new applications to maintain the results.

## CONCLUSION

In this study, laser therapy was found to be a positive and safe treatment option for Harlequin syndrome, yielding favorable outcomes in terms of both clinical symptoms and signs and patient perception. These findings suggest that laser therapy could be a viable procedure for improving the aesthetic and quality-of-life aspects of patients with Harlequin syndrome. Furthermore, the positive effects of this novel treatment serve as a valuable foundation for future research focusing on developing alternative approaches to effectively enhance the clinical management of this condition.

## Data Availability

The underlying content is contained within the manuscript.
