# Supplementary material for: Laser therapy: palliative care for the Harlequin syndrome?
Source: Einstein (Sao Paulo). 2026 Feb 2;24:eRC1544. doi: 10.31744/einstein_journal/2026RC1544 (PMC12977264; doi:10.31744/einstein_journal/2026RC1544)
Supplement: SUPPLEMENTARY MATERIAL [file 2317-6385-eins-24-eRC1544-suppl01.pdf]

SUPPLEMENATRY MATERIAL

Laser therapy: palliative care for the Harlequin syndrome?

Felipe Otávio Saraiva França, Guilherme Albuquerque Nicolau, Mariana Machado Lima, Rodrigo Otávio Gama França, Vania Flavia Siqueira Saraiva

DOI: 10.31744/einstein_journal/2026RC1544

Table 1S. Questionário baseado no modelo TSQM, utilizado para avaliação da eficácia do tratamento

Quão satisfeita ou insatisfeita você está com a capacidade do procedimento de tratar sua condição?
Muito Satisfeita
Quão satisfeita ou insatisfeita você está com a forma como o procedimento alivia seus sintomas?
Extremamente Satisfeita
Quão satisfeita ou insatisfeita você está com o tempo que leva para o procedimento começar a fazer efeito?
Extremamente Satisfeita
Quão desconfortável é a realização do procedimento?
Não senti desconforto no procedimento
No geral, quão confiante você está de que esse procedimento fez bem para você?
Muito confiante
Quão certa você está de que as coisas boas sobre o tratamento superam as coisas ruins?
Muito confiante
Levando tudo em consideração, quão satisfeito ou insatisfeito você está com este medicamento?
Extremamente Satisfeito
